# LPS induces microglial activation and GABAergic synaptic deficits in the hippocampus accompanied by prolonged cognitive impairment

**DOI:** 10.1038/s41598-023-32798-9

**Published:** 2023-04-21

**Authors:** Hyeji Jung, Dongsu Lee, Heejung You, Myungha Lee, Hyeonho Kim, Eunji Cheong, Ji Won Um

**Affiliations:** 1 Department of Brain Sciences, Daegu Gyeongbuk Institute of Science and Technology (DGIST), 333 Techno Jungangdae-Ro, Hyeonpoong-Eup, Dalseong-Gun, Daegu, 42988 Korea; 2 Department of Biotechnology, College of Life Science and Biotechnology, Yonsei University, Seoul, 03722 Korea; 3 Center for Synapse Diversity and Specificity, DGIST, 333 Techno Jungangdae-Ro, Hyeonpoong-Eup, Dalseong-Gun, Daegu, 42988 Korea

**Keywords:** Microglia, Neurotransmitters, Molecular biology, Neuroscience

## Abstract

Neuroinflammation impacts the brain and cognitive behavior through microglial activation. In this study, we determined the temporal sequence from microglial activation to synaptic dysfunction and cognitive behavior induced by neuroinflammation in mice. We found that LPS injection activated microglia within a short period, followed by impairments in GABAergic synapses, and that these events led to long-term cognitive impairment. We demonstrated that, 3 days after LPS injection, microglia in the hippocampus were significantly activated due to the LPS-induced inflammation in association with alterations in cellular morphology, microglial density, and expression of phagocytic markers. GABAergic synaptic impairments were detected at 4–6 days after LPS treatment, a time when microglia activity had returned to normal. Consequently, memory impairment persisted for 6 days after injection of LPS. Our results suggest that neuroinflammation induces microglia activation, GABAergic synaptic deficits and prolonged memory impairment over a defined temporal sequence. Our observations provide insight into the temporal sequence of neuroinflammation-associated brain pathologies. Moreover, the specific loss of inhibitory synapses accompanying the impaired inhibitory synaptic transmission provides mechanistic insight that may explain the prolonged cognitive deficit observed in patients with neuroinflammation. Thus, this study provides essential clues regarding early intervention strategies against brain pathologies accompanying neuroinflammation.

## Introduction

Neuroinflammation is a complex combination of acute and chronic responses of cells within the central nervous system initiated by various stimuli, including infection, traumatic brain injury, toxic metabolites, and autoimmunity^1^. It has been proposed that neuroinflammation contributes to the initiation and progression of neurodegenerative diseases, including Alzheimer’s disease (AD), and Parkinson’s disease, as well as neuropsychiatric disorders such as schizophrenia and depression^2,3^. Microglia, the resident macrophages in the brain, are the primary responders and play critical roles in the onset and progression of neuroinflammation^4^. Microglia are rapidly activated during neuroinflammation, undergoing molecular and cellular changes that manifest as synaptic deficits, neuronal cell loss, altered levels of neurotransmitters and/or oxidative stress^4^.

Controlling the excitability, dynamic range, input gating, and neural coding across a variety of neural circuits requires a balance between excitation and inhibition^5^. An imbalance between excitation and inhibition in neural circuits is considered a leading cause of various neurological disorders^6^. In particular, alterations in the excitation–inhibition (E–I) balance in the hippocampal CA1 region is a critical pathological mechanism underlying dysregulated memory formation^7^. One of the significant functions of microglia in the central nervous system (CNS) is shaping synapses through synapse elimination, a process mediated by phagocytosis. It was initially reported that microglia contribute to the reconstruction of neuronal circuits through phagocytosis of excess neuronal synapses and newborn neurons during brain development via the classical complement pathway^8–11^. Recent studies showed that microglia are actively involved in synapse dynamics in the brains of healthy, adult mice^12,13^, contributing to normal memory extinction and sleep. In addition, microglia have been shown to eliminate synapses using the complement cascade in disease conditions^14–16^. Although activated microglia influence the number, morphology and/or connections of synapses under the neuroinflammatory conditions^17–21^, the temporal sequences of cellular and behavioral changes induced by microglia-associated neuroinflammation remain elusive.

We previously showed that two daily intraperitoneal injections of 0.5 mg/kg lipopolysaccharide (LPS) activate microglia globally, including in the hippocampus^22^. In the current study, we utilized the same neuroinflammation model, in conjunction with imaging, electrophysiology and behavioral assays, to demonstrate neuroinflammation-induced temporal changes in microglia, synapses, and cognition. In particular, to deduce the relationship between changes in synaptic function and memory-related behavioral changes, we focused on cellular changes in hippocampal CA1 regions of the brain, which are well known for their roles in memory^23–25^. We demonstrate that microglial activation is triggered transiently by LPS administration, followed by GABAergic synaptic alterations. Cognitive deficits induced by LPS administration were prolonged, despite the fact that microglial activation had returned to normal.

## Materials and methods

### Animals

C57BL/6J mice used in the current study were maintained and handled in accordance with protocols (DGIST-IACUC-20122401-0004 and Yonsei-IACUC-A-202201-1405-02) approved by the Institutional Animal Care and Use Committee (IACUC) of the Daegu Gyeongbuk Institute of Science and Technology (DGIST) or Yonsei University. Mice were maintained on a 12:12-h light: dark cycle under standard, temperature (22 ± 2 °C) -controlled laboratory conditions, and received water and food ad libitum. All experimental procedures were performed on male mice and were conducted according to the guidelines and protocols for rodent experimentation approved by the IACUC of DGIST and ARRIVE guidelines^26^.

### Antibodies

The following commercially available antibodies were used: rabbit polyclonal anti-Iba-1 (Fujifilm Wako, Cat# 016-20,001; RRID: AB_839506), rat monoclonal anti-CD68 (clone FA-11; Bio-Rad, Cat# MCA1957GA; RRID: AB_324217), rabbit polyclonal anti-GFAP (ABFrontier, Cat# AR09-PA0002; RRID: AB_2755016), rabbit polyclonal anti-VGAT (Synaptic Systems, Cat# 131 003; RRID: AB_887869), rabbit polyclonal anti-GABA_A_Rγ2 (Synaptic Systems, Cat# 224 003; RRID: AB_2263066), mouse monoclonal anti-gephyrin (Synaptic Systems, Cat# 147 111; RRID: AB_887719), mouse monoclonal anti-GAD67 (Millipore, Cat# MAB5406; RRID:AB_2278725), mouse monoclonal anti-PV (Swant, Cat# 235; RRID: AB_10000343), rat monoclonal anti-SST (Millipore, Cat# MAB354; RRID: AB_2255365), mouse monoclonal anti-β-actin (Santa Cruz, Cat# sc-47778; RRID: AB_626632), mouse monoclonal anti-VGAT (Synaptic Systems, Cat# 131 011; RRID: AB_887872), and guinea pig polyclonal anti-VGLUT1 (Millipore, Cat# AB5905; RRID: AB_2301751). The following antibodies have been previously described: anti-PSD-95 (JK016; RRID: AB_2722693) and anti-VGLUT1 (JK111; RRID: AB_2810945)^27^.

### LPS injection and immunohistochemistry

Eight-week-old mice were subjected to two daily intraperitoneal injections of 0.5 mg/kg LPS (from *Escherichia coli* O111:B4, L3012, Sigma, Lot# 12 170 308) or phosphate-buffered saline (PBS) at a 24-h-interval. LPS was first dissolved in distilled water, diluted to a final concentration of 0.05 mg/mL with PBS, and injected into mice in a volume of 10 μL per gram of body weight. At different time points after the final injection (3, 4, 6, or 8 d), mice were deeply anaesthetized by inhalation of isoflurane and immediately perfused, first with PBS for 3 min and then with 4% paraformaldehyde for 5 min. Brains were dissected out, fixed in 4% paraformaldehyde overnight, dehydrated in 30% sucrose in PBS for at least 3 days, and sliced into 40-μm-thick coronal sections using a cryotome (Model CM-3050-S; Leica Biosystems). Brain sections were permeabilized by incubating with 0.2% Triton X-100 in PBS containing 5% bovine serum albumin and 5% horse serum for 1 h at room temperature. For immunostaining, brain sections were incubated for 16 h at 4 °C with primary anti-Iba-1 (1:400), anti-CD68 (1:300), anti-GFAP (1:300), anti-VGLUT1 (1:300), anti-PSD-95 (1:300), anti-VGAT (1:500), anti-GABA_A_Rγ2 (1: 1000), anti-gephyrin (1:600), anti-GAD67 (1:300), anti-PV (1:500), or anti-SST (1:100) antibodies diluted in the same blocking solution. Sections were washed three times in PBS and incubated with appropriate Cy3 or fluorescein isothiocyanate (FITC)-conjugated secondary antibodies (Jackson ImmunoResearch) for 2 h at room temperature. After three washes with PBS, sections were mounted onto glass slides (Superfrost Plus; Fisher Scientific) with Vectashield mounting medium (H-1200; Vector Laboratories). For anti-gephyrin antibody staining, a heating-based antigen-retrieval procedure using citrate buffer (10 mM sodium citrate, 0.05%[v/v] Tween-20, pH 8.0) preceded permeabilization of sections. Images were acquired by confocal microscopy (LSM800 or LSM700; Zeiss). Synaptic puncta were quantified using MetaMorph software (Molecular Devices), and their density and average size were measured. Neuron somata immunopositive for GAD67, PV, and/or SST were quantified manually.

### Analysis of microglia morphology

Z-stack images were acquired at 3-μm intervals over a 15-μm Z-range using a confocal microscope (Zeiss LSM800) and then converted into single-plane maximal intensity projection images using Zen2.6 Software (Zeiss). Microglia within Z-projection images were counted, contoured manually, and analyzed with respect to their size and degree of co-localization of Iba-1-positive (Iba-1^+^) and CD68^+^ signals, expressed as a percentage (Iba-1^+^CD68^+^ area/Iba-1^+^ area × 100%) using MetaMorph software (Molecular Devices). Microglia in Z-projection images were categorized as ramified cells, with small/round soma and highly arborized long/thin processes (3-times longer than the length of soma); bushy cells, with enlarged cell bodies and swollen/retracted processes (less than 3-times, but more than 1.5-times longer than the length of soma); and amoeboid cells, with condensed cell bodies with few or no process (processes shorter than the length of soma).

For skeleton analyses, all Iba-1 immunostaining maximal intensity projection images were first enhanced to visualize microglia processes under the same condition, after which images were converted to binary images, skeletonized and analyzed using the AnalyzeSkeleton plugin (http://imagejdocu.tudor.lu/) in Image J software, as previously described^28^.

### Quantification of 3D microglial engulfment

For analysis of microglial engulfment, Z-stack images were acquired at 1-μm intervals over a 20-μm Z-range using a confocal microscope (Zeiss LSM800) with a 40 × objective. Images were analyzed with Imaris software (BitPlane) by three-dimensionally reconstituting Iba-1^+^ microglia using the "Surface" function. The resulting microglial surface rendering was used to mask the VGAT/gephyrin/VGLUT1 channel so as to define the engulfed synaptic puncta within microglial surface. VGAT^+^, gephyrin^+^ or VGLUT1^+^ synaptic puncta within microglial surfaces were detected and quantified using the "Spots" function. The number of synaptic puncta within each microglial cell was normalized to the volume of the microglia.

### Preparation of brain lysates and immunoblot analysis

A P2 (crude synaptosome) subcellular fraction was prepared as previously described^27^. Protein concentration was measured by using the Bradford assay, and samples were immunoblotted with the antibodies indicated in each figure legends. Western blot images were quantified using ImageJ software (National Institutes of Health).

### Behavioral analysis

*1. Open-field test.* Mice were placed in the test room for at least 30 min for habituation and then were placed in white-colored acrylic chamber (40 × 40 × 40 cm; length × width × height) for 30 min at 50 lx of light intensity with white noise to assess general locomotor activity and anxiety. The traveled distance and time spent in the center zone by freely moving mice were recorded by a top-view infrared camera and analyzed using EthoVision XT 10 software (Noldus). *2. Novel object-recognition test.* An open-field chamber was used in this test. For training sessions, two identical objects were placed in the center of the chamber at regular intervals, and mice were allowed to explore the objects for 5 min. After the training session, mice were returned to their home cage for 5 min. For novel object-recognition tests, one of the two objects was replaced with a new object, placed in the same position in the chamber. Mice were returned to the chamber and allowed to explore freely for 5 min. The movement of mice was recorded by infrared camera, and the number and duration of contacts were analyzed using EthoVision XT 10 (Noldus). For the analysis of novel object preferences, the time to explore each object during the test period was measured for each mouse and preference was calculated using the following equation: Discrimination index (DI) = (novel object exploration time − control object exploration time)/(total exploration time). Mice that explore the objects for less than 2 s during training phase or testing phase were not included in this study^29–32^. First 3 min out of 5 min test phase were quantified. *3. Light and dark transition test.* The light/dark box used consisted of a roofless, bright chamber conjoined to a closed, dark chamber. A small entrance allowed free travel between the two chambers. The light chamber was illuminated at 360 lx. The time spent in each chamber was measured. The movement of mice was recorded with white noise by a top-view infrared camera and analyzed using EthoVision XT 10.5 (Noldus).

### Whole-cell patch-clamp recordings

Male C57BL/6 J mice after NORT test were anesthetized with halothane and decapitated to remove the brains. The brains were quickly transferred and sectioned in an oxygentaed (95% O_2_/5% CO_2_) ice-cold artificial cerebrospinal fluid solution (aCSF: 130 mM NaCl, 3.5 mM KCl, 24 mM NaHCO_3_, 1.25 mM NaH_2_PO_4_, 1.5 mM MgCl_2_-H_2_O, 1.5 mM CaCl_2_-H_2_O, and 10 mM D-glucose). Horizontal slices (300 μm) were prepared using a vibrating-knife microtome VT1000s (Leica Microsystems, Germany). Slices were stabilized for at least 1 h in an oxygenated aCSF. Slices were transferred to the recording chamber and perfused in an oxygenated aCSF. Individual cells were visualized using an upright Olympus EX51WI (Olympus, Japan) microscope equipped with an ORCA-R2 camera (Hamamatsu, Japan). Patch electrodes (2–5 MΩ) were prepared with capillary glass (Warner Instruments, USA) using a micropipette puller (P-97, Sutter Instrument, CA). Intra-pipette solution containing (in mM): 130 CsCl, 2 MgCl_2_-H_2_O, 10 HEPES, 5 ATP, 0.5 Na-GTP and 0.1 EGTA. Additionally, 5 mM QX-314 (Sigma) was used for the measurement of spontaneous and evoked inhibitory postsynaptic currents (sIPSCs and eIPSCs). For experiments in which inhibitory postsynaptic currents (IPSCs) were recorded, currents were pharmacologically isolated with bath application of 50 µM APV (Tocris Bioscience), and 50 µM CNQX (Sigma). The hippocampal CA1 pyramidal neurons in pyramidal layer were held at − 70 mV. Extracellular stimulation of local neurons within specific layers of the hippocampus was achieved by current injection through a stimulating electrode placed in the relevant layer within 100–200 μm. Signals were amplified using MultiClamp 700B (Molecular Devices, USA), and data acquisition was performed using a DigiData 1550B (Axon Instruments) and Clampex (Molecular Devices, USA). All data analyses were performed using the Clampfit 10.6 (Molecular Devices, USA) and Mini Analysis software (Synaptosoft Inc.). The amplitude of eIPSCs was calculated by averaging the 20 sweeps (excluding failures). Paired-pulse ratios were calculated by recording the amplitudes of paired eIPSC with 100 ms stimulation interval.

### Statistical analysis

All data are presented as means ± standard error of the mean (SEM). The normality of data distributions was evaluated using the D'Agostino & Pearson test. Data were statistically evaluated using a Mann–Whitney *U* test, ordinary one-way analysis of variance (ANOVA) followed by with post hoc Tukey’s test, Dunnett test, or Kruskal–Wallis test, as appropriate. Prism8.0 (GraphPad Software) was used for analysis of data and preparation of bar graphs. *P*-values < 0.05 were considered statistically significant.

### Ethics approval

All procedures and protocols were approved by the Institutional Animal Care and Use Committee of Daegu Gyeongbuk Institute of Science and Technology (DGIST) (protocol number: DGIST-IACUC-20122401-0004) or Yonsei University (Protocol number: Yonsei-IACUC-A-202201-1405-02). All experiments were conducted according to the guidelines and protocols for rodent experimentation approved by the Institutional Animal Care and Use Committee of DGIST or Yonsei University.

## Results

### Sickness behaviors induced by LPS injection persist for four days in mice

**Figure 1:**
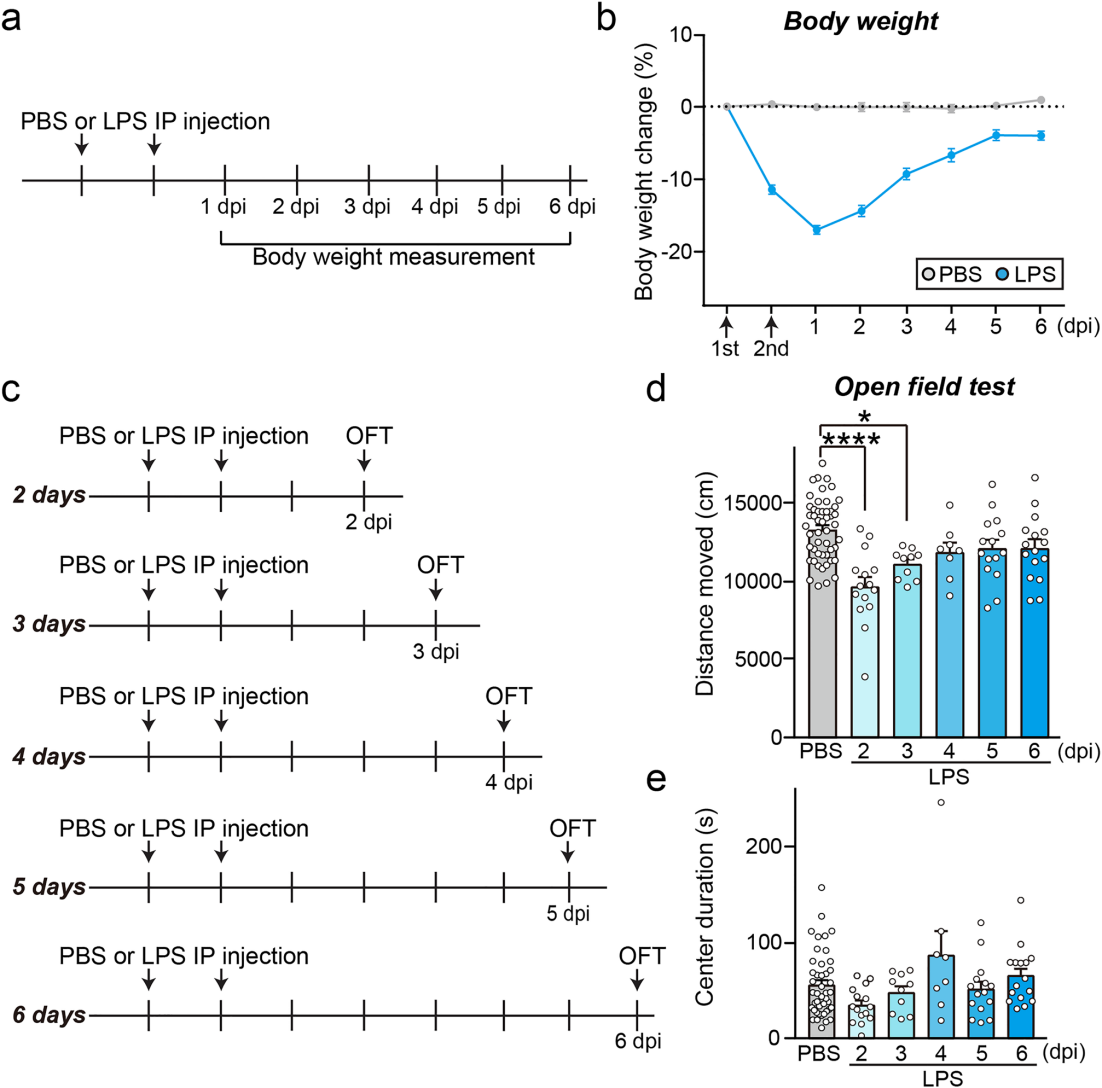
Behavioral signs of sickness under systemic inflammation are alleviated by day 4 after LPS injection in mice. (**a**) Schematic illustration of the experimental procedure for performing LPS injection and daily body-weight measurement of mice. (**b**) The LPS-injected mice initially displayed a body weight reduction of about 20% body at 1 dpi, and body width recovered to 90% by day 4 after LPS injection. Each measured body weight was normalized by that obtained before the first injection. The level of 1 is marked by a dotted line (PBS, N = 14; LPS N = 22). (**c**) Schematic illustration showing the procedures used for LPS administration and the open-field test (OFT). (**d**, **e**) Quantification of the distance moved (**d**) and center duration (**e** in OFT conducted 2–6 days after injection of LPS or PBS (****P < 0.0001, *P < 0.05; PBS, N = 50; LPS, N = 8–16; one-way ANOVA with post-hoc Dunnett’s test).

First, to investigate the effect of neuroinflammation on cognitive functions, we induced systemic inflammation by intraperitoneal injection of LPS. It has been reported that systemic administration of LPS produces a spectrum of acute behavioral responses termed sickness behavior and neuroinflammation with reactive glial cells^33–37^. However, how long sickness behavior and microglia-mediated neuroinflammation persistently influence the animals’ behavior needs to be described. To monitor the duration of this acute phase sickness behavior, we injected mice intraperitoneally with LPS once a day for 2 d, then checked baseline parameters such as locomotor activity and body weight daily until they recovered back to normal range. We observed that the LPS injected groups displayed a significant body weight loss of about 20% right after LPS injection and recovered back to 90% four days after LPS injection, parallel with the recovery of locomotor activity observed in the open field test (OFT) (Fig. 1).

### Microglia are transiently activated in the hippocampus of the LPS-injected mice

**Figure 2:**
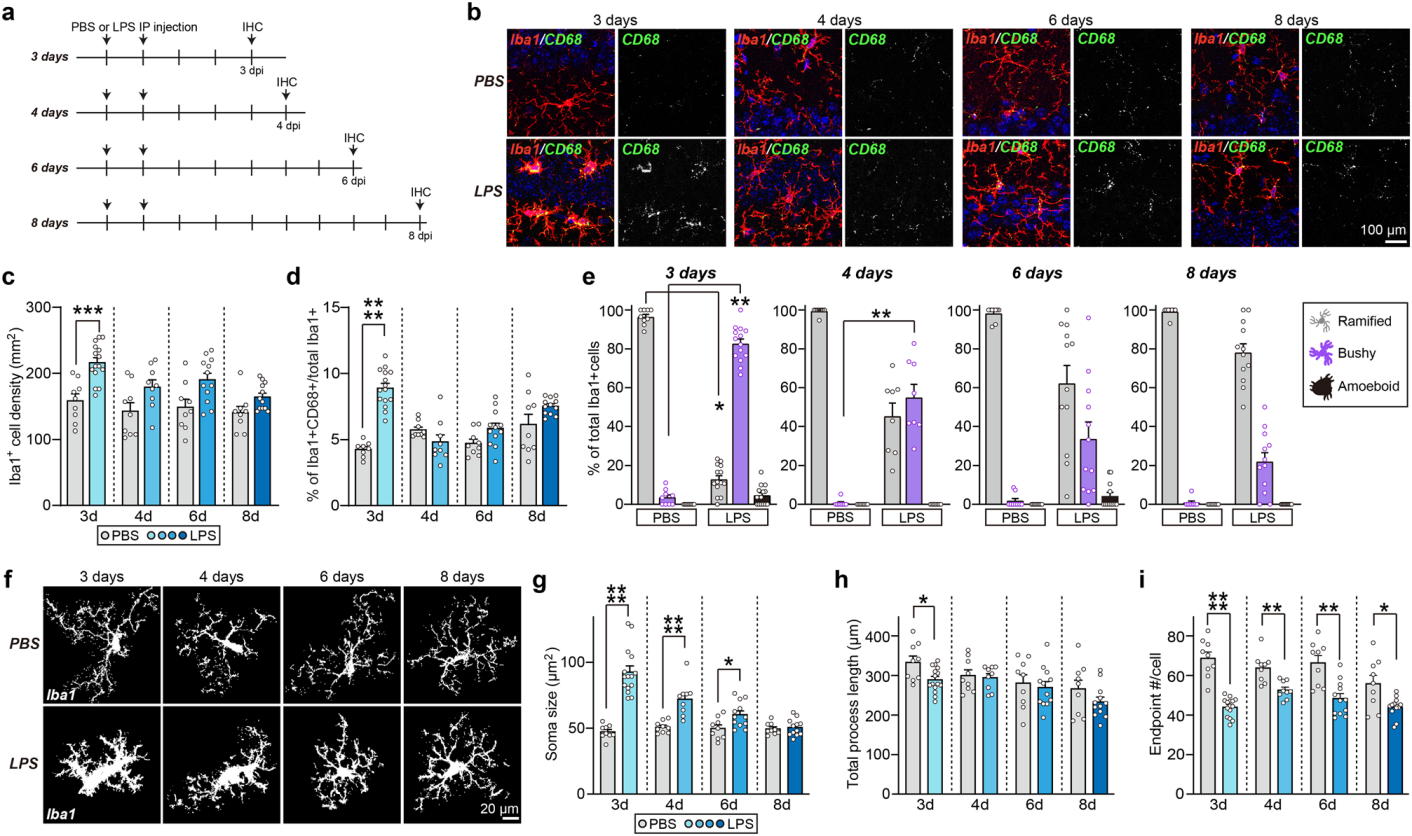
LPS treatment induces a transient increase in microglial activity and number in the hippocampus of adult mice. (**a**) Schematic illustration of the experimental procedure for LPS administration and immunohistochemistry (IHC) in mice. C57BL/6J male mice at the age of 8 wk were intraperitoneally (i.p.) injected with 0.5 mg/kg LPS or an equal volume of saline daily for 2 d. IHC was performed at 3, 4, 6, and 8 d post-LPS injection (dpi). (**b**) Representative images of hippocampal CA1 regions of mouse brains 3, 4, 6, and 8 d after LPS treatment. Brain sections were immunostained for Iba-1 (red) and CD68 (green). Scale bars: 100 μm (applies to all images). (**c**) Quantification of the density of Iba-1^+^ cells. Data are means ± SEMs (***P < 0.001; Ordinary one-way ANOVA with Tukey’s test; n = 8–15 slices from 3 to 5 mice). (**d**) Quantification of the colocalization percentage of Iba-1^+^/CD68^+^ cells. Data are means ± SEMs (****P < 0.0001; Ordinary one-way ANOVA with Tukey’s test; n = 8–15 slices from 3 to 5 mice). (**e**) Percentage of microglia displaying a ramified, bushy or amoeboid morphology. Data are means ± SEM (**P < 0.01; *P < 0.05 vs. Control group; Kruskal–Wallis test; n = 8–15 slices from 3 to 5 mice). (**f**) Representative binary images of Iba-1 staining in hippocampal CA1 regions of mouse brains 3, 4, 6, and 8 d after LPS treatment. Scale bars: 20 μm (applies to all images). (**g**) Quantification of the size of Iba-1^+^ cell bodies. Data are means ± SEMs (****P < 0.0001; *P < 0.05; Mann–Whitney *U*-test; n = 9–15 slices from 3 to 5 mice per group). (**h**) Quantification of the total lengths of Iba-1^+^ cells processes. Data are means ± SEMs (*P < 0.05; Mann–Whitney *U*-test; n = 9–15 slices from 3 to 5 mice per group). (**i**) Quantification of the number of Iba-1^+^ cell process endpoints. Data are means ± SEMs (****P < 0.0001; **P < 0.01; *P < 0.05; Mann–Whitney *U*-test; n = 9–15 slices from 3 to 5 mice per group).

To examine the temporal changes in microglia activation by LPS, we performed an immunohistochemical assessment of the numbers of microglia positive for the microglial marker, Iba-1 (ionized calcium-binding adapter molecule-1) and the expression levels of CD68, a lysosomal protein that is frequently used as a marker for actively phagocytic microglia^38^ at 3, 4, 6, and 8 d post-injection (dpi) (Fig. 2a). We observed a significant elevation in microglia numbers and CD68 expression at 3 dpi in the hippocampal CA1 regions, but this increase did not persist and was absent at 4–8 dpi (Fig. 2b–d). We then analyzed the morphology of microglia, as it is known to correlate well with their activation status^39^. Immunohistochemical analyses revealed morphological changes in microglia from ramified to a bushy form at 3 dpi, followed by a slow reversion towards normal at 4–6 dpi and recovery toward normal at 8 dpi (Fig. 2e). In addition, an analysis of soma size, total length of microglia processes, and the number of endpoints of microglia revealed that soma size of microglia was increased at 3 dpi, followed by a gradual decrease towards normal; the total length of microglia processes was significantly decreased only at 3 dpi; and the number of endpoints was significantly decreased at 3–8 dpi showing the biggest difference at 3 dpi and smallest difference at 8 dpi (Fig. 2f-i). Astrocyte activation was not observed at 3, 4, 6, and 8 dpi, as determined by immunostaining with an anti-GFAP antibody (Fig. S1). These results suggest that microglial activation occurred by 3 dpi and that the microglial state subsequently normalized starting from 4 dpi.

### The number of GABAergic synapses is subsequently decreased following LPS-induced microglial activation

**Figure 3:**
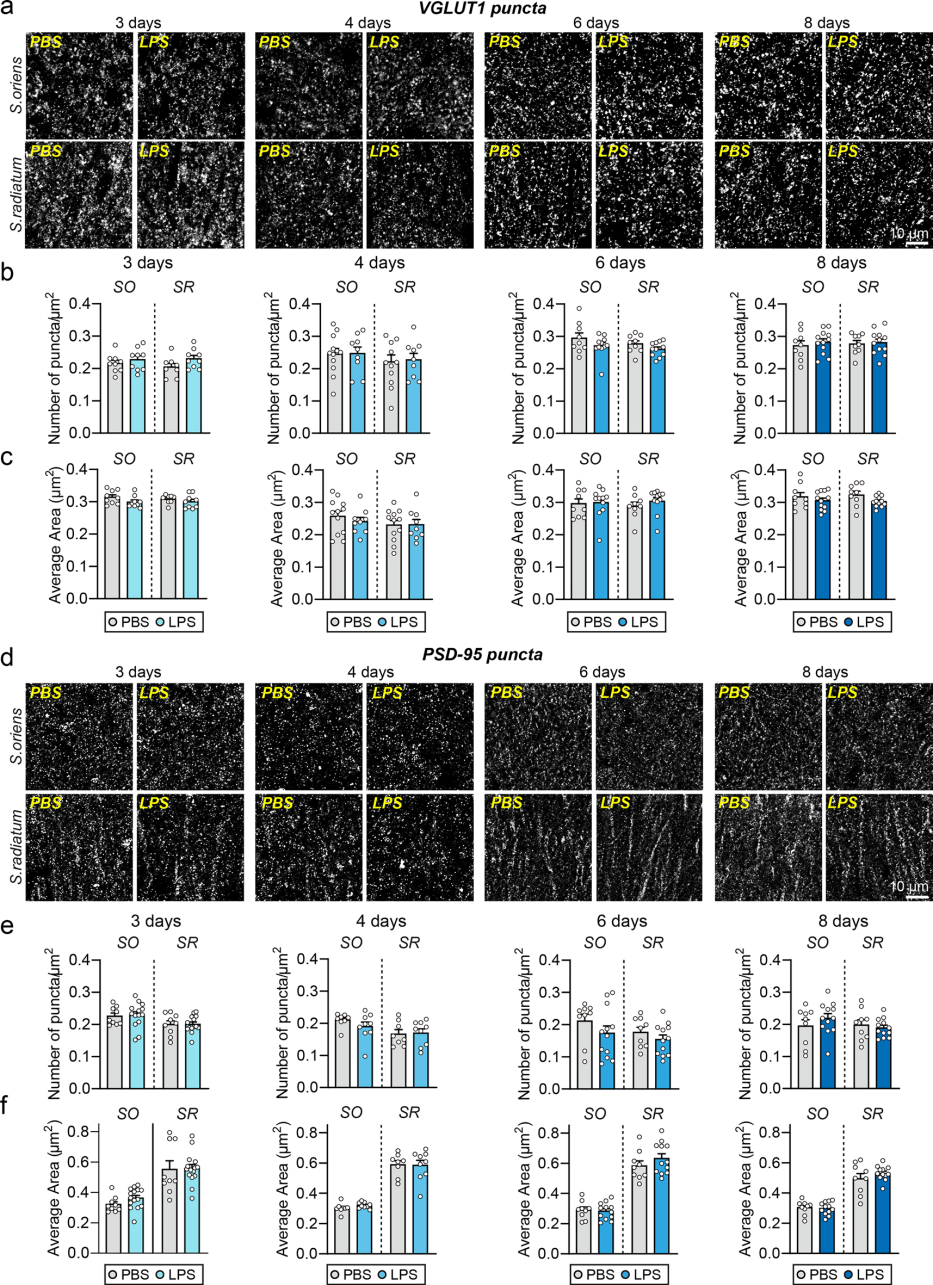
LPS-induced neuroinflammation has no effect on the maintenance of excitatory synapses in the hippocampus of adult mice. (**a**, **d**) Representative images of hippocampal CA1 *SO*, and *SR* layers 3, 4, 6, and 8 d after LPS administration in mice, followed by immunostaining for the excitatory synapse marker VGLUT1 (**a**) or PSD-95 (**d**). Scale bar: 10 μm (applies to all images). (**b**, **c**, **e**, **f**) Quantification of the density and area of VGLUT1-positive (**b**, **c**) or PSD-95–positive (**e**, **f**) synaptic puncta. Data are means ± SEMs (n = 8–15 slices from 3 to 5 mice per group).

**Figure 4:**
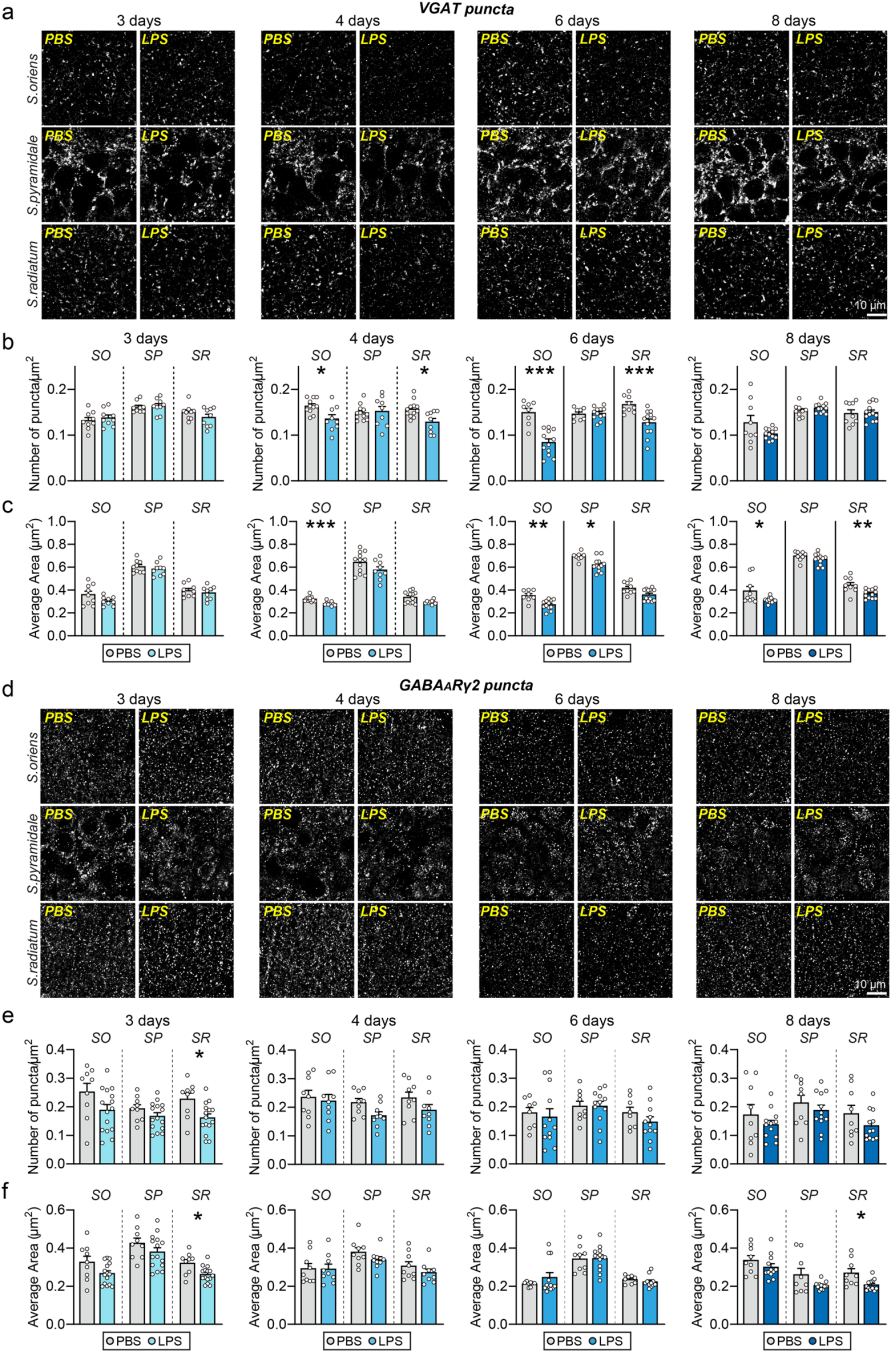
Decreased GABAergic synapse numbers are preceded by LPS-induced microglial activation. (**a**, **d**) Representative images of hippocampal CA1 *SO*, *SP* and *SR* layers 3, 4, 6, or 8 days after LPS administration in mice, followed by immunostaining for the inhibitory synapse marker VGAT (**a**) or GABA_A_Rγ2 (**d**). Scale bar: 10 μm (applies to all images). (**b**, **c**, **e**, **f**) Quantification of the density and area of VGAT-positive (**b**, **c**) or GABA_A_Rγ2-positive (**e**, **f**) synaptic puncta. Data are means ± SEMs (*P < 0.05; **P < 0.01; ***P < 0.001; Mann–Whitney *U*-test; n = 8–15 slices from 3 to 5 mice per group).

Next, to investigate whether LPS-induced microglial activation contributed to synaptic alterations, we analyzed excitatory and inhibitory synaptic puncta in various layers of the hippocampal CA1 region at 3, 4, 6, and 8 dpi. Quantitative immunofluorescence analyses of excitatory synaptic markers showed that the density and average size of VGLUT1 (vesicular glutamate transporter 1; excitatory presynaptic marker) and PSD-95 (post-synaptic density 95 kDa; excitatory postsynaptic marker) puncta were unchanged in all examined layers of hippocampal CA1 regions (stratum oriens [SO], stratum radiatum [SR], stratum pyramidale [SP]) at all time points in LPS-treated mice (Fig. 3). In contrast, the density and average size of VGAT (vesicular GABA transporter; inhibitory presynaptic marker) puncta were significantly decreased in hippocampal CA1 regions at 4 dpi and 6 dpi, while minor changes were seen in the density and average area of GABA_A_Rγ2 (inhibitory postsynaptic marker) puncta (Fig. 4). These data indicate that neuroinflammation specifically induces alterations in GABAergic presynaptic number and size, and that LPS-induced microglial activation at 3 dpi was followed by a reduction in GABAergic presynapses at 4–6 dpi, an effect that also returned to normal at 8 dpi.

**Figure 5:**
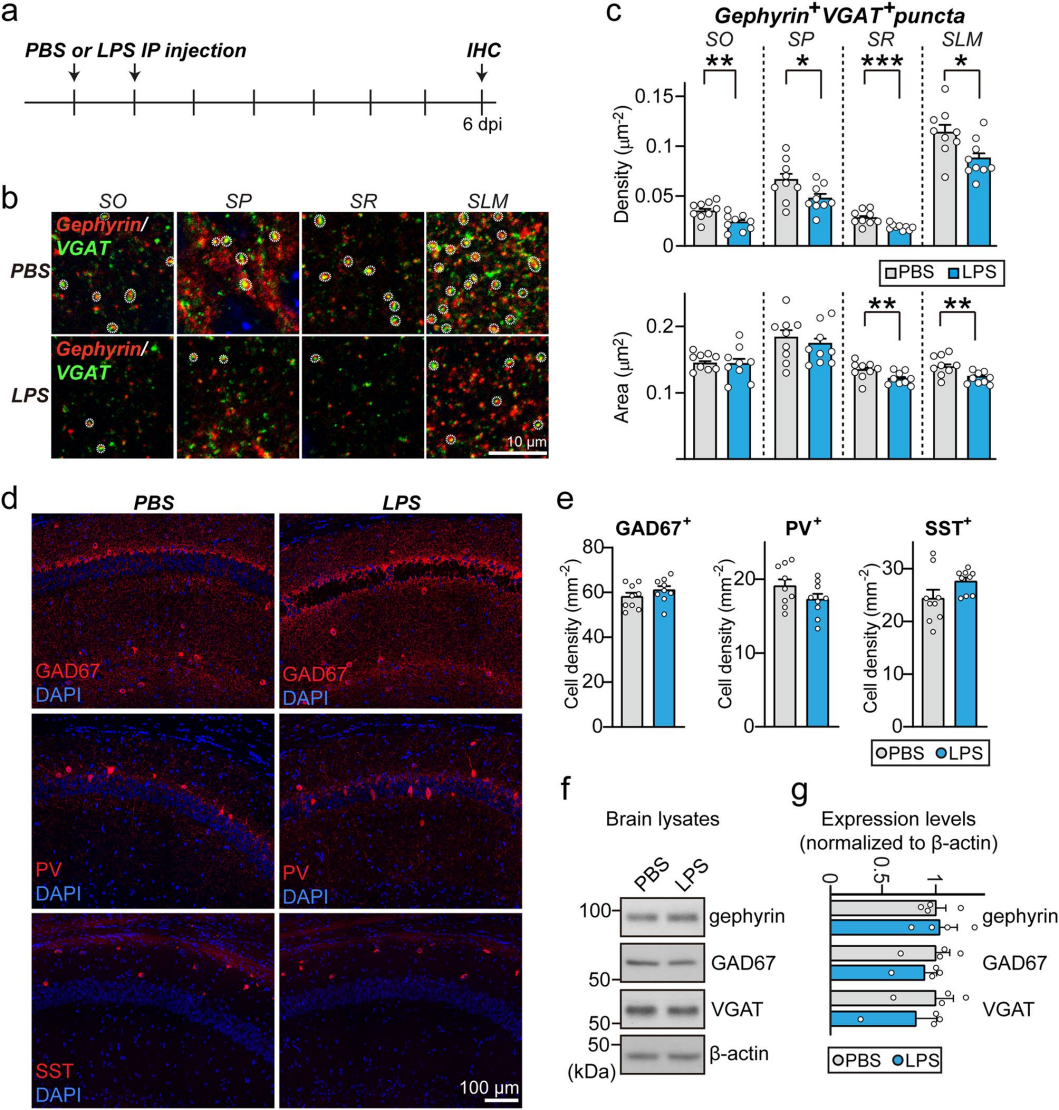
LPS-induced neuroinflammation leads to impaired maintenance of GABAergic synapses without alterations in the number of GABAergic interneurons or the levels of GABAergic synaptic proteins. (**a**) Schematic illustration of the experimental procedure for LPS administration and immunohistochemistry (IHC) in mice. (**b**) Representative images of hippocampal CA1 *SO*, *SP*, *SR* and *SLM* layers 6 d after LPS treatment. Brain sections were immunostained for gephyrin (red) and VGAT (green). White circles mark colocalization of gephyrin with VGAT puncta. Scale bars: 10 μm (applies to all images). (**c**) Quantification of the density and area of gephyrin^+^VGAT^+^ synaptic puncta. Data are means ± SEMs (*P < 0.05; **P < 0.01; ***P < 0.001; Mann–Whitney *U*-test; n = 9 slices each from 3 mice). (**d**) Representative images of hippocampal CA1 regions 6 d after LPS treatment. Brain sections were immunostained for GAD67, PV or SST. Scale bars: 100 μm (applies to all images). (**e**) Quantification of the density of GAD67^+^, PV^+^, or SST^+^ interneurons. Data are means ± SEMs (n = 9 slices each from 3 mice). (**f**, **g**) Representative immunoblots (**f**) of hippocampal lysates, and summary data showing GABAergic synaptic protein levels (**g**) in crude synaptosomal fractions of PBS and LPS treated mice, analyzed by semi-quantitative immunoblotting. Data are means ± SEMs (n = 4 mice/group; Mann–Whitney *U*-test).

To further confirm the effect of neuroinflammation on GABAergic synapses, we performed semiquantitative immunohistochemical analyses using anti-VGAT and anti-gephyrin antibodies at 6 dpi. VGAT/gephyrin double-positive inhibitory puncta density was significantly reduced in all examined layers of hippocampal CA1 regions (SO, SP, SR and stratum lacunosum moleculare [SLM]) in LPS-treated mice (Fig. 5a–c). The reduction in GABAergic synaptic components was not attributable to the reduction of GABAergic interneurons or the changes in protein levels because the number of GABAergic interneurons in the hippocampus CA1 regions (Fig. 5d,e) or the protein levels of gephyrin, VGAT or GAD67 (Glutamic acid decarboxylase 67) (Fig. 5f,g) were not reduced at 6 dpi.

### Microglial engulfment of GABAergic presynapses is increased in the hippocampus of LPS-injected mice

**Figure 6:**
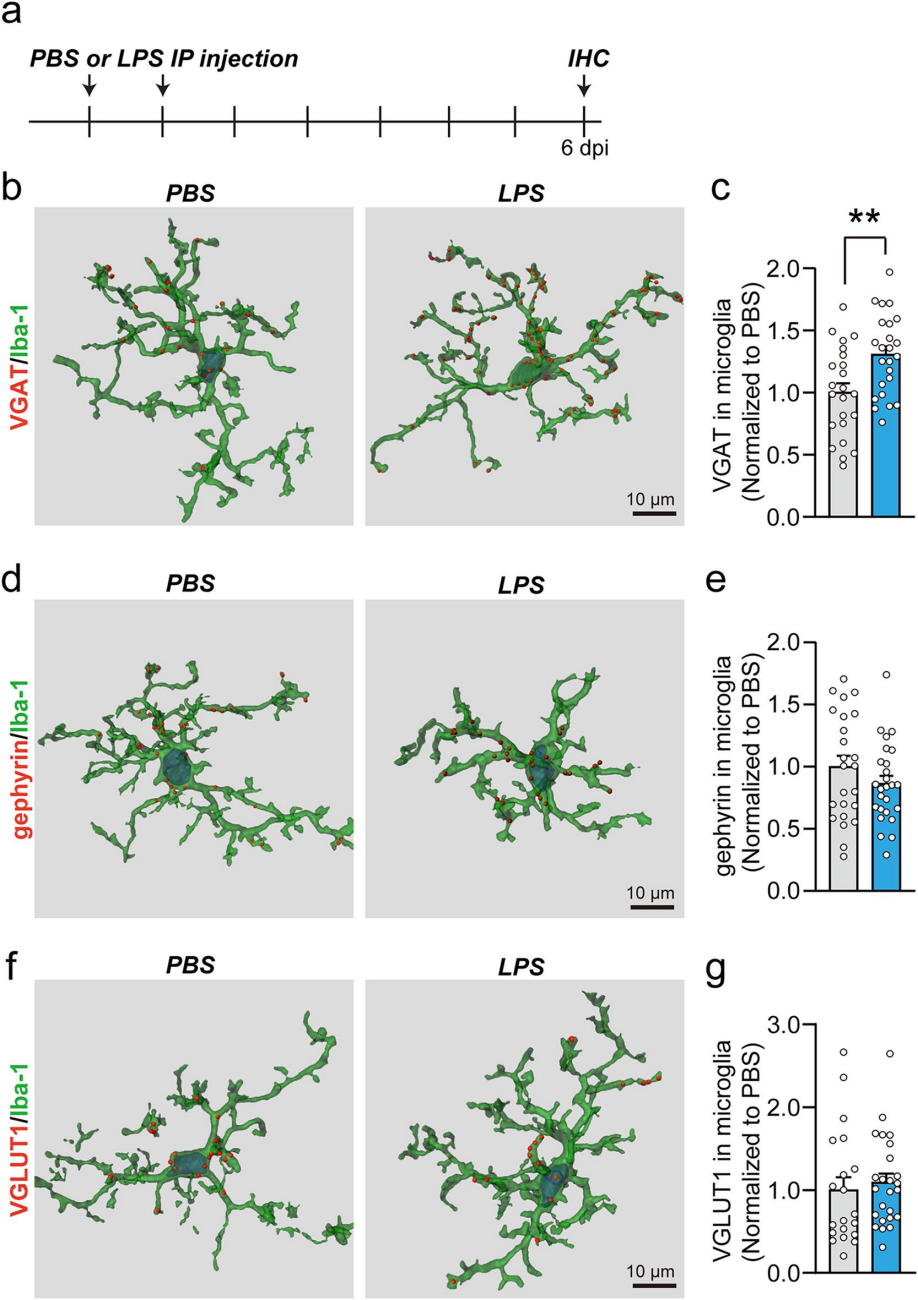
Neuroinflammation drives increased microglial engulfment of GABAergic presynapses. (**a**) Schematic illustration of the experimental procedure for LPS administration and immunohistochemistry (IHC) in mice. (**b**, **d**, **f**) Representative 3-D surface-rendering images. (**c**, **e**, **g**) Quantification of the number of VGAT puncta (**c**), gephyrin puncta (**e**) or VGLUT1 puncta (**g**) inside Iba-1^+^ microglia in the hippocampal CA1 SO layer. Data are means ± SEMs (**P < 0.01; Mann–Whitney *U*-test; n = 20–29 cells from 2 slices of 3 mice per group).

Microglia exhibit phagocytic activity, which is critical for synapse pruning and removal of dead cells^9,11^. Accordingly, we examined whether the decreased number of GABAergic synapses observed in LPS-treated mice is attributable to microglial engulfment. To this end, we performed an immunofluorescence analysis to evaluate the number of VGAT-positive (VGAT^+^), gephyrin^+^ or VGLUTI^+^ puncta within Iba-1^+^ microglia in hippocampal CA1 regions (Fig. 6a). Confocal imaging coupled with 3D cell surface rendering revealed that microglia in LPS-treated mice contained significantly more VGAT^+^ puncta than microglia from PBS-treated mice (Fig. 6b,c) whereas there was no significant difference in the number of gephyrin + puncta within microglia between LPS-treated mice and control mice (Fig. 6d,e). In addition, the number of VGLUTI^+^ puncta within microglia was unchanged in LPS-treated mice (Fig. 6f,g). These data suggest that the specific loss of GABAergic synapses observed in LPS-treated mice is likely caused by microglia-mediated engulfment of GABAergic presynapses.

### Impaired short-term memory induced by neuroinflammation is correlated with GABAergic synaptic transmission deficits

**Figure 7:**
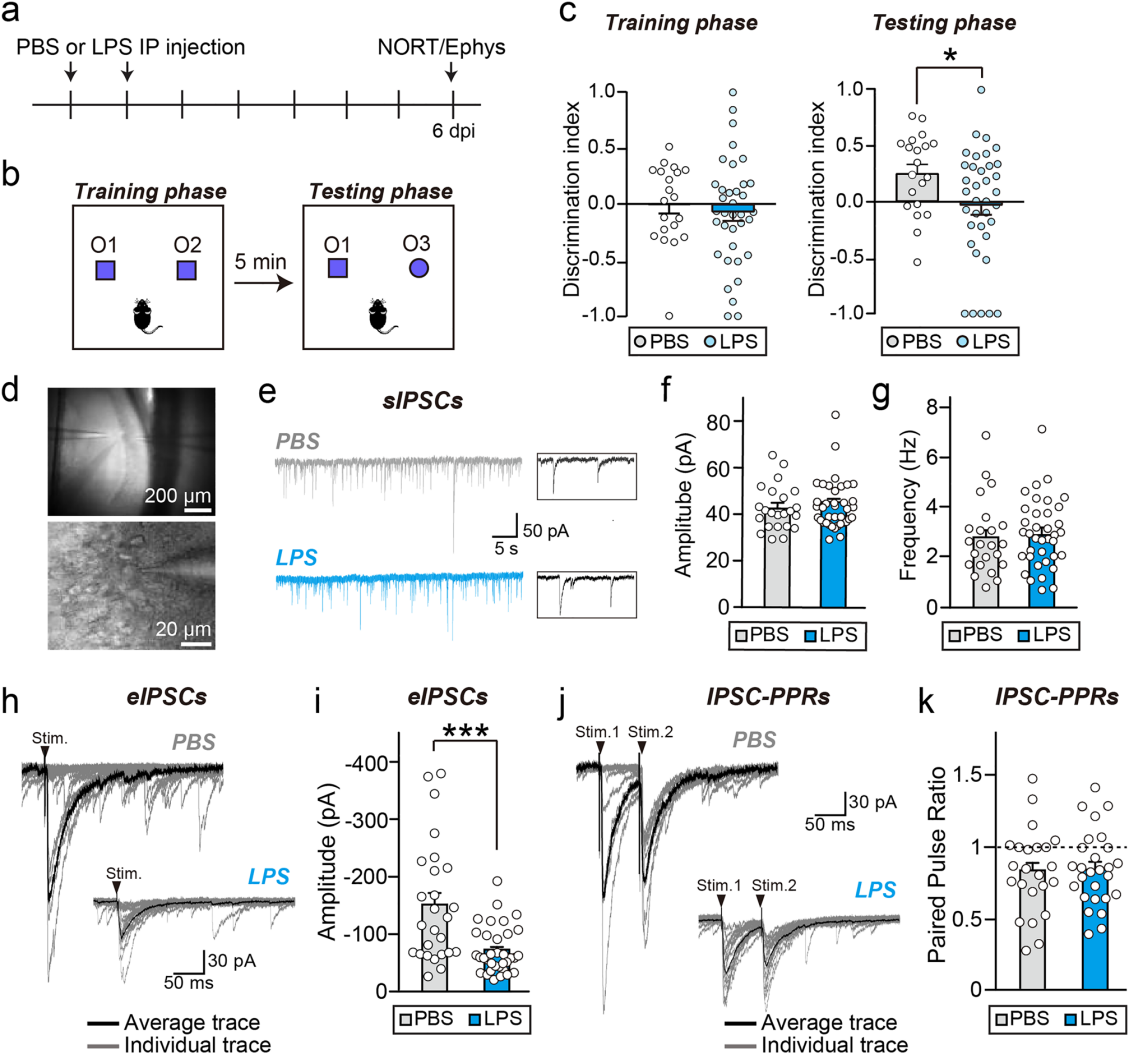
Impaired short-term memory induced by neuroinflammation is correlated with GABAergic synaptic transmission deficits. (**a**) Schematic illustration of the experimental procedure for LPS administration, novel object recognition test (NORT), followed by electrophysiological recordings (ephys) on hippocampal neurons ex vivo in mice. (**b**) Design of experiments measuring novel object-recognition memories. Two identical objects (O1 and O2) were used for the training phase, and one familiar object (O1) and one novel object (O3) were used for the testing phase. (**c**) Discrimination index in the training phase (left) or testing phase (right) was plotted. Data are presented as means ± SEMs (training phase: PBS, 0 ± 0.08; LPS, − 0.01 ± 0.08; *P* = 0.5358; testing phase: PBS, 0.25 ± 0.08; LPS, − 0.03 ± 0.08; *P < 0.05; PBS, N = 20; LPS, N = 36; Mann–Whitney *U*-test). (**d**) Bright field images showing a whole-cell recording from a hippocampal CA1 pyramidal neuron. (**e**) Representative spontaneous IPSC traces (left) and enlarged trace (right) of hippocampal CA1 pyramidal neurons treated with PBS versus LPS. (**f**, **g**) The amplitudes (**f**) and frequencies (**g**) of sIPSCs recorded from hippocampal slices of mice treated with LPS or PBS. Data are presented as means ± SEMs (amplitude: PBS, 43.74 ± 1.87; LPS, 45.48 ± 1.66; *P* = 0.5360; frequency: PBS, 2.80 ± 0.31; LPS, 2.96 ± 0.22; *P* = 0.4873; PBS, n = 23 from 18 mice; LPS, n = 37 from 14 mice; Mann–Whitney *U*-test). (**h**) Representative evoked IPSC (eIPSC) traces of hippocampal CA1 pyramidal neurons. The individual trace of each electrical stimulation is shown in gray and the average traces obtained from 20 iterations are shown in black. (**i**) The amplitudes of eIPSCs recorded from hippocampal slices was significantly smaller for mice treated with LPS compared to those treated with PBS (amplitude: PBS, − 151.49 ± 20.30; LPS, − 72.13 ± 7.03; ***P = 0.0002; PBS, n = 26 from 18 mice; LPS, n = 34 from 14 mice; Mann–Whitney *U*-test). (**j**, **k**) There was no difference between PBS- and LPS-treated groups in the paired-pulse ratio (PPR) obtained with various stimulus intervals (PPR: PBS, 0.83 ± 0.06; LPS, 0.85 ± 0.05; *P* = 0.9692; PBS, n = 23 from 18 mice; LPS n = 27 from 14 mice; Mann–Whitney *U*-test).

Previous studies showed that the altered E-I balance induced by reduced GABAergic synapses in the hippocampus induces cognitive and memory deficits in mice. Hence, we tested whether the neuroinflammation-induced impairment of GABAergic synapses could impact mouse behaviors. We performed behavioral tests on mice at 6 dpi, as we wished to capture the significant decrease in GABAergic synapses in CA1 regions seen between 4 and 6 dpi (Fig. 4), but exclude any sickness behavior lasting up to ~ 4 dpi (Fig. 1). We found that PBS- and LPS-treated mice at 6 dpi did not differ in their overall locomotor activity measured in the open-field test (Fig. 1) or anxiety-related behavior in the light and dart transition test (Fig. S2). However, in the novel object-recognition test, in which mice were exposed to two identical objects in the training session and then to one new object and one familiar object in the testing session, the LPS-treated mice exhibited a severe memory deficit (signaled by a significantly lower discrimination index) compared with PBS-treated mice at 6 dpi (Fig. 7a-c). To examine whether this LPS-induced memory deficit was correlated with impaired GABAergic synapse functions, we performed whole-cell electrophysiological recordings in acute hippocampal slices obtained from LPS- and PBS-treated mice at 6 dpi after a novel object-recognition test. There was no difference in the amplitude or frequency of spontaneous inhibitory postsynaptic currents (sIPSCs) recorded from CA1 hippocampal neurons of LPS-treated mice compared to PBS-treated mice (Fig. 7d-g). Interestingly, the amplitudes of evoked inhibitory postsynaptic currents (eIPSCs) stimulated by axons in the SP layer were significantly decreased (Fig. 7h,i) without alteration of the paired-pulse ratio (Fig. 7j,k). Collectively, these data suggest that the neuroinflammation induces explicit alterations in the structure and function of GABAergic synapses at 6 dpi, resulting in a memory deficit that persists even after alleviation of the acute phase of neuroinflammation and microglial activation.

## Discussion

Although numerous studies have identified neuroinflammation as a common etiopathogenic factor in various neurological disorders, including AD, and synapse loss is considered an early hallmark of neurological disorders^3,40,41^, the sequence of cellular events and the duration of the effects on cognitive behaviors had remained unknown. We demonstrate here that microglia are activated within a short time after LPS administration, followed by GABAergic synapse deficits, and that such events can result in long-lasting cognitive impairment.

The current study showed that LPS-induced inflammation resulted in significant activation of microglia in the hippocampus, with associated changes in cellular morphology, numbers, and expression of phagocytic markers by 3 dpi. In particular, we extensively analyzed several morphological parameters of microglia including soma size, total length of processes and endpoint numbers at various time points after LPS administration, given that previous studies have established that morphological changes are correlated with hyperactivation of microglia^20,21^. In the present study, all these parameters showed the biggest difference in response to LPS at 3 dpi and gradually returned towards normal at 6–8 dpi. We detected GABAergic synaptic deficits beginning at 4 dpi, when microglial activity appeared to have returned to its typical level; these deficits persisted up to 8 dpi (Fig. 4 and 5), suggesting that the synaptic alteration had long-lasting effects. Our results therefore show that, in this model, microglial activation precedes the synapse loss, which is explicitly manifested as a reduction in the number of GABAergic synapses. These data are consistent with previous studies reporting that microglia-mediated synaptic sculpting during neuroinflammation mainly targets GABAergic synapses^18,42^. Our data also pointed out that the cognitive deficits measured within 48 to 72 h after systemic LPS administration reported in previous studies^43–45^ may be primarily due to sickness behavior, as the structural and functional changes in the neural circuits do not appear until 4 dpi after LPS administration.

The persistent structural alteration of GABAergic synapses agreed well with the memory impairment seen at 6 dpi after LPS treatment. Moreover, mice with memory impairment exhibited significantly reduced eIPSC amplitude. These results together imply that the structural changes in GABAergic synapses (Fig. 4 and 5) correlate with alteration in the functional synaptic transmissions at GABAergic synapses and the memory deficits observed in LPS-injected mice (Fig. 7). Neuronal excitability or activity is regulated by the combinatorial integration of excitatory synaptic inputs and balancing GABAergic synaptic inputs, which directs appropriate behaviors. Our study indicates that GABAergic synaptic deficits might affect hippocampal neuronal activity, leading to impaired memory function. This idea is keeping with previous studies showing that spontaneous or sensory-driven neuronal activity is impaired during peripheral inflammation^46^. Given that intracellular Ca^2+^ signaling in microglia is altered during peripheral inflammation^47^, it might be informative to investigate whether activity among neurons and microglia is synchronized or otherwise linked. Many neurological disorders can induce neuroinflammation, including infections, stroke, trauma, stress, and autoimmune diseases. In addition, microglial activation precedes synapse loss, specifically manifesting as a reduction in the number of GABAergic synapses. Our data collectively suggest that microglia-mediated GABAergic synapse loss may contribute to alteration in the synaptic E-I balance in the hippocampus and subsequently affect memory function in mice. In particular, altered E-I balance induced by a reduction in GABAergic synapses in the hippocampus was shown to result in cognitive and memory deficits in mice. Mice with a deletion of IQSEC3 or collybistin, key GABAergic synaptic components, exhibit deficits in hippocampus-dependent spatial learning or fear memory formation, respectively^27,48^. Moreover, impairment in APP-mediated GABAergic synapse signaling by the negative regulator MDGA1 (MAM domain-containing glycosylphosphatidylinositol anchor 1) in hippocampal CA1 regions also impairs novel object-recognition memory in mice^25^. In the present study, we focused on cellular changes in the hippocampus, but the LPS dose employed in this study caused microglial activation to different extents not only in the hippocampus but in a variety of brain regions^22^. Hence, microglial activation-induced synaptic/circuit changes in different brain regions might lead to alteration in corresponding aspects of behavior and cognitive functions in mice, and contribute to subsequent progression of specific, relevant neurological diseases. Future studies are necessary to assess whether the temporal sequence of biological processes identified in this study is similarly recapitulated in other brain regions and address whether synaptic/circuit changes in other brains regions also induce specific behavioral deficits.

Then, how GABAergic synapses are specifically affected by LPS-induced microglial activation should also be further determined in future studies. Activated microglia might directly interact with and eliminate GABAergic presynaptic components through microglial phagocytosis via a classical complement cascade; alternatively, pro/anti-inflammatory cytokines released from activated microglia might indirectly interact with their own receptors expressed in neurons and transduce signal cascades that regulate GABAergic synapses. In the present study, we observed that LPS treatment significantly increased engulfment of GABAergic presynapse by microglia in vivo (Fig. 6), suggesting that the loss of GABAergic synapse in the LPS-treated mice could be caused by microglia-mediated synapse elimination rather than by pro/anti-inflammatory cytokines released from activated microglia. This result is in line with previous studies reporting the involvement of microglia in pathological synapse loss and dysfunction following injury, inflammation, or nervous system degeneration^34,49–51^. The molecular mechanisms by which GABAergic presynapses are specifically engulfed by microglia remain to be determined.

In sum, we determined the temporal sequences of neuroinflammation-induced cellular changes in the hippocampus and hippocampal-dependent behavioral alterations that may be linked to neuroinflammation-associated brain malfunctions or pathologies. Our study provides strong evidence that heightened neuroinflammation acts as an important factor that may contribute to and exacerbate cognitive decline, and provides important clues regarding early intervention strategies against pathologies associated with neuroinflammation.

## Supplementary Information

Supplementary Information.

## Data Availability

The data sets analyzed during the current study are available from the corresponding authors on reasonable request.

## References

[1] Sochocka M, Diniz BS, Leszek J (2017). Inflammatory response in the CNS: Friend or foe?. Mol. Neurobiol..

[2] Matta SM, Hill-Yardin EL, Crack PJ (2019). The influence of neuroinflammation in Autism spectrum disorder. Brain Behav. Immun..

[3] Leng F, Edison P (2021). Neuroinflammation and microglial activation in Alzheimer disease: Where do we go from here?. Nat. Rev. Neurol..

[4] Aguzzi A, Barres BA, Bennett ML (2013). Microglia: Scapegoat, saboteur, or something else?. Science.

[5] Bhatia A, Moza S, Bhalla U (2019). U. S. Precise excitation-inhibition balance controls gain and timing in the hippocampus. eLife.

[6] O’Donnell C, Gonçalves JT, Portera-Cailliau C, Sejnowski TJ (2017). Beyond excitation/inhibition imbalance in multidimensional models of neural circuit changes in brain disorders. eLife.

[7] Nanou E, Lee A, Catterall WA (2018). Control of excitation/inhibition balance in a hippocampal circuit by calcium sensor protein regulation of presynaptic calcium channels. J. Neurosci..

[8] Tremblay M-È (2011). The role of microglia in the healthy brain. J. Neurosci..

[9] Paolicelli RC (2011). Synaptic pruning by microglia is necessary for normal brain development. Science.

[10] Stevens B (2007). The classical complement cascade mediates CNS synapse elimination. Cell.

[11] Schafer DP (2012). Microglia sculpt postnatal neural circuits in an activity and complement-dependent manner. Neuron.

[12] Wang C (2020). Microglia mediate forgetting via complement-dependent synaptic elimination. Science.

[13] Choudhury ME (2020). Phagocytic elimination of synapses by microglia during sleep. Glia.

[14] Lui H (2016). Progranulin deficiency promotes circuit-specific synaptic pruning by microglia via complement activation. Cell.

[15] Vasek MJ (2016). A complement–microglial axis drives synapse loss during virus-induced memory impairment. Nature.

[16] Sekar A (2016). Schizophrenia risk from complex variation of complement component 4. Nature.

[17] Chen Z (2012). Lipopolysaccharide-induced microglial activation and neuroprotection against experimental brain injury is independent of hematogenous TLR4. J. Neurosci..

[18] Chen Z (2014). Microglial displacement of inhibitory synapses provides neuroprotection in the adult brain. Nat. Commun..

[19] Cao P (2021). Early-life inflammation promotes depressive symptoms in adolescence via microglial engulfment of dendritic spines. Neuron.

[20] Gyoneva S (2014). Systemic inflammation regulates microglial responses to tissue damage in vivo. Glia.

[21] Kozlowski C, Weimer RM (2012). An automated method to quantify microglia morphology and application to monitor activation state longitudinally in vivo. PloS One.

[22] Jung H (2022). Differential regional vulnerability of the brain to mild neuroinflammation induced by systemic LPS treatment in mice. J. Inflamm. Res..

[23] Tsien JZ, Huerta PT, Tonegawa S (1996). The essential role of hippocampal CA1 NMDA receptor-dependent synaptic plasticity in spatial memory. Cell.

[24] Bartsch T, Döhring J, Rohr A, Jansen O, Deuschl G (2011). CA1 neurons in the human hippocampus are critical for autobiographical memory, mental time travel, and autonoetic consciousness. Proc. Natl. Acad. Sci..

[25] Kim J (2022). MDGA1 negatively regulates amyloid precursor protein–mediated synapse inhibition in the hippocampus. Proc. Natl. Acad. Sci..

[26] du Percie SN (2020). Reporting animal research: Explanation and elaboration for the ARRIVE guidelines 20. PLoS Biol..

[27] Kim D (2022). IQSEC3 deletion impairs fear memory through upregulation of ribosomal S6K1 signaling in the hippocampus. Biol. Psychiat..

[28] Young K, Morrison H (2018). Quantifying microglia morphology from photomicrographs of immunohistochemistry prepared tissue using imageJ. JoVE.

[29] Reger ML, Hovda DA, Giza CC (2009). Ontogeny of Rat Recognition Memory measured by the novel object recognition task. Dev. Psychobiol..

[30] Kwak H (2020). Astrocytes control sensory acuity via tonic inhibition in the thalamus. Neuron.

[31] Vogel-Ciernia A, Wood MA (2014). Examining object location and object recognition memory in mice. Curr. Protocols Neurosci..

[32] Balasco L (2022). Abnormal whisker-dependent behaviors and altered cortico-hippocampal connectivity in Shank3b−/− mice. Cereb. Cortex.

[33] Kent S, Bluthé R-M, Kelley KW, Dantzer R (1992). Sickness behavior as a new target for drug development. Trends Pharmacol. Sci..

[34] Savage JC, St-Pierre M-K, Hui CW, Tremblay M-E (2019). Microglial ultrastructure in the hippocampus of a lipopolysaccharide-induced sickness mouse model. Front. Neurosci..

[35] Ifuku M (2012). Anti-inflammatory/anti-amyloidogenic effects of plasmalogens in lipopolysaccharide-induced neuroinflammation in adult mice. J. Neuroinflammation.

[36] Wendeln A-C (2018). Innate immune memory in the brain shapes neurological disease hallmarks. Nature.

[37] Krishna S, Dodd CA, Filipov NM (2016). Behavioral and monoamine perturbations in adult male mice with chronic inflammation induced by repeated peripheral lipopolysaccharide administration. Behav. Brain Res..

[38] Park D (2020). Seizure progression triggered by IQSEC3 loss is mitigated by reducing activated microglia in mice. Glia.

[39] Sierra A, Paolicelli RC, Kettenmann H (2019). Cien Años de Microglía: Milestones in a century of microglial research. Trends Neurosci..

[40] Kinney JW (2018). Inflammation as a central mechanism in Alzheimer's disease. Alzheimer's Dementia.

[41] Selkoe DJ (2002). Alzheimer's disease is a synaptic failure. Science.

[42] Li S-M (2020). A complement-microglial axis driving inhibitory synapse related protein loss might contribute to systemic inflammation-induced cognitive impairment. Int. Immunopharmacol..

[43] Frühauf PKS (2015). Spermine reverses lipopolysaccharide-induced memory deficit in mice. J. Neuroinflammation.

[44] Liu L (2020). Nrf2 deficiency exacerbates cognitive impairment and reactive microgliosis in a lipopolysaccharide-induced neuroinflammatory mouse model. Cell. Mol. Neurobiol..

[45] Mastinu A (2019). Gamma-oryzanol prevents LPS-induced brain inflammation and cognitive impairment in adult mice. Nutrients.

[46] Odoj K (2021). In vivo mechanisms of cortical network dysfunction induced by systemic inflammation. Brain Behav. Immun..

[47] Riester K (2020). In vivo characterization of functional states of cortical microglia during peripheral inflammation. Brain Behav. Immun..

[48] Papadopoulos T (2007). Impaired GABAergic transmission and altered hippocampal synaptic plasticity in collybistin-deficient mice. EMBO J..

[49] Hong S, Dissing-Olesen L, Stevens B (2016). New insights on the role of microglia in synaptic pruning in health and disease. Curr. Opin. Neurobiol..

[50] Xin Y-R (2019). The immune system drives synapse loss during lipopolysaccharide-induced learning and memory impairment in mice. Front. Aging Neurosci..

[51] Werneburg S (2020). Targeted complement inhibition at synapses prevents microglial synaptic engulfment and synapse loss in demyelinating disease. Immunity.

