# Supplementary material for: LPS induces microglial activation and GABAergic synaptic deficits in the hippocampus accompanied by prolonged cognitive impairment
Source: Sci Rep. 2023 Apr 21;13:6547. doi: 10.1038/s41598-023-32798-9 (PMC10121592; doi:10.1038/s41598-023-32798-9)
Supplement: Supplementary file 1 — Supplementary Information. [file 41598_2023_32798_MOESM1_ESM.pdf]

Supplementary information

LPS induces microglial activation and GABAergic synaptic deficits in the hippocampus accompanied by prolonged cognitive impairment

Jung et al.

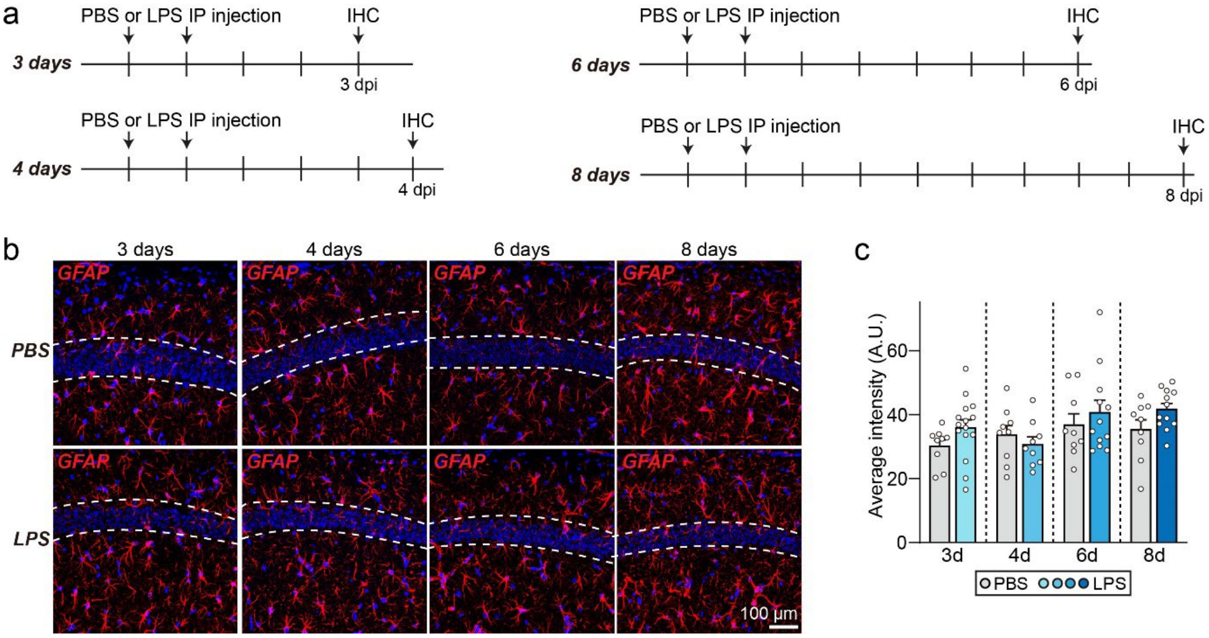

Fig. S1. LPS treatment induces a transient astrogliosis in the hippocampus of adult mice.

a Schematic illustration of the experimental procedure for LPS administration and immunohistochemistry (IHC) in mice. C57BL/6 male mice at the age of 8 wk were intraperitoneally (i.p.) injected with 0.5 mg/kg LPS or an equal volume of saline daily for 2 d. IHC was performed at 3, 4, 6, and 8 d post-LPS injection (dpi). **b** Representative images of hippocampal CA1 regions of mouse brains 3, 4, 6, and 8 d after LPS treatment. Brain sections were immunostained for GFAP (red). Scale bars: 100 μm (applies to all images). **c** Quantification of the average intensity of $GFAP^{+}$ cells. Data are means ± SEMs (n = 9–15 slices from 3–5 mice).

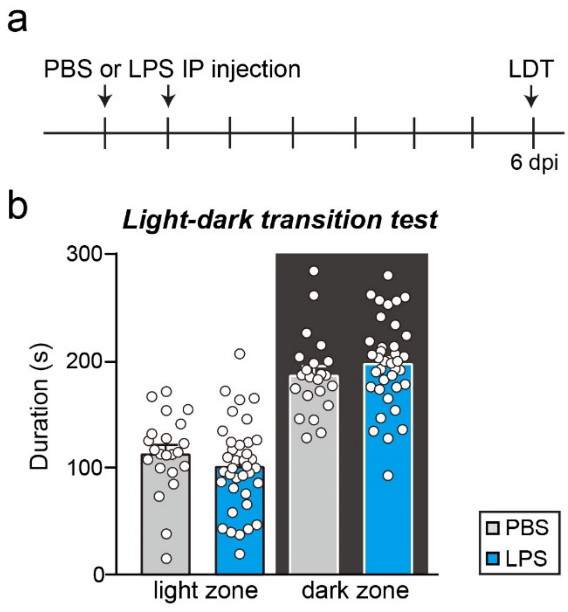

Figure S2. Anxiety-like behavior was not altered in LPS-injected mice.

a Schematic illustration of the experimental procedure for performing intraperitoneal injection of LPS or PBS and applying the light-dark transition test in mice. **b** The LPS-injected mice displayed normal anxiety-like behavior, displaying a preference for the dark zone. There was no difference between PBS- and LPS-injected mice in the time spent in the dark zone (light zone: PBS, 113.05 ± 7.97; LPS, 186.95 ± 7.97; $p = 0.1405$; dark zone: PBS, 101.67 ± 6.80; LPS, 198.33 ± 6.80; $p = 0.1405$; PBS, N = 22; LPS, N = 37; Mann-Whitney *U*-test).

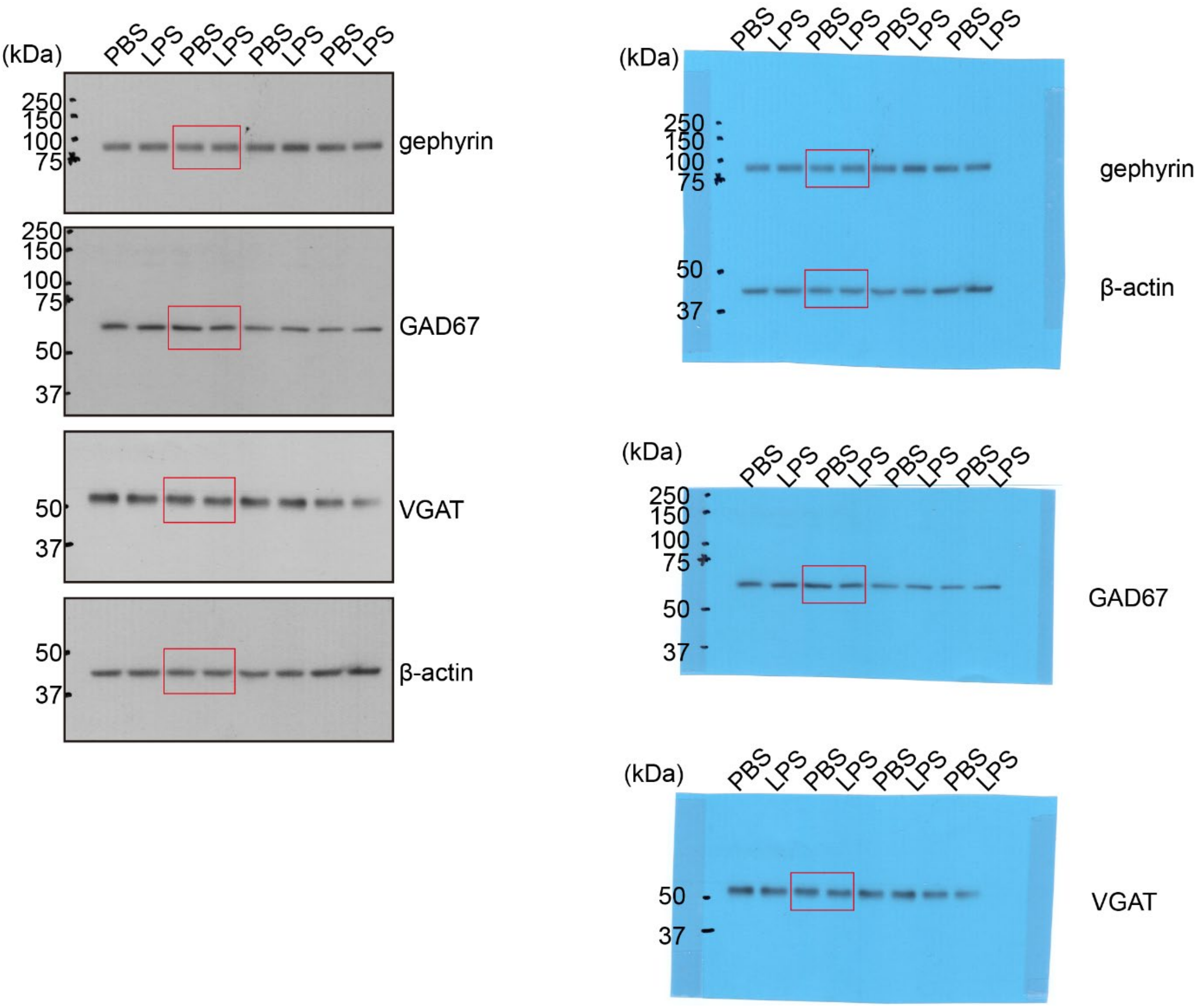

Figure S3. Uncropped WB images.

The original WB images used for Figure 5F. Red boxes indicate cropped lines.
